# The Effect of Night-Time Feeding on Steer Performance After Terminal Sort

**DOI:** 10.3390/ani16121912

**Published:** 2026-06-20

**Authors:** Madeline R. Mancke, Brad J. White, Eduarda M. Bortoluzzi, Robert L. Larson

**Affiliations:** 1 Beef Cattle Institute, Kansas State University, Manhattan, KS 66506, USArlarson@k-state.edu (R.L.L.); 2 Department of Anatomy and Physiology, Kansas State University, Manhattan, KS 66506, USA; bortoluzzi@vet.k-state.edu

**Keywords:** feedyard, heat mitigation, heat stress, night-time feeding, steers

## Abstract

Heat stress occurs when total environmental and metabolic heat production is greater than an animal’s ability to dissipate that heat. Heat stress negatively impacts feeder cattle performance and welfare. Limited research has been conducted to determine if feeding cattle in the evening, thereby shifting their metabolic heat production to a cooler period of the day, can help mitigate heat stress. This pen-level randomized controlled trial evaluated the effects of evening feeding (PM; feedings at 2000, 2300, and 0200 h; *n* = 24 pens) versus morning feeding (AM; feedings at 0500, 0800, and 1200 h; *n* = 24 pens) on terminally sorted steer performance in a commercial feedyard in the Pacific Northwest. Data collection included feed delivery, water consumption, health events, open mouth breathing prevalence, and carcass traits. Linear and generalized linear mixed-effects models were used to determine potential differences between treatment group and temperature-humidity index (THI; <80 versus ≥80). Only 14% of the total study days had a THI ≥ 80, indicating little to no heat stress impacts. There were no differences found between PM and AM for any outcome (*p* < 0.05). Regardless of treatment group, water consumption tended (*p* = 0.07) to increase, and open mouth breathing significantly (*p* < 0.05) increased on days with THI ≥ 80. Further research is warranted to assess evening feeding as a heat stress mitigation strategy in a feedyard setting.

## Simple Summary

This randomized controlled trial evaluated the effects of evening feeding on mitigating heat stress and improving performance in feedyard steers. At a commercial feedyard in the Pacific Northwest, a total of 7848 terminally sorted steers were assigned to either morning (AM; 0500, 0800, 1200 h) or evening (PM; 2000, 2300, 0200 h) feeding schedules. Outcomes measured included water consumption, feed delivery, health events, open mouth breathing behavior, and carcass traits. The temperature-humidity index was used to assess heat stress conditions. A limited number of study days (14%) reached THI ≥ 80, indicating generally mild environmental conditions. No significant treatment differences were detected for performance, health, behavior, or carcass outcomes. Regardless of feeding schedule, increased THI was associated with increased water consumption and a greater prevalence of open mouth breathing, indicating some effects of heat stress. Further research during periods of greater environmental stress is warranted to determine the effectiveness of evening feeding as a heat stress mitigation strategy.

## 1. Introduction

Heat stress occurs when total heat accumulation (both environmental and metabolic) exceeds the animal’s ability to dissipate heat [1,2]. Cattle respond to high environmental temperatures behaviorally, physiologically, and cellularly in an attempt to maintain homeostasis [3,4]. In extreme situations, hyperthermia can result in a large number of deaths [5]. In a review published by Lees et al. in 2019 [6], authors associated heat stress with reductions in dry matter intake, growth, and feed conversion efficiency in feedyard cattle. Additionally, an increase in respiration rate, panting score, and body temperature are commonly observed during times of heat stress [7,8,9]. In 2003, St-Pierre estimated that there was a $370 million (USD) economic burden on the beef industry attributable to heat stress [10]. Given that this estimate is over 20 years old, the current economic burden is likely higher today.

Today, cattle are grown to greater body weights and spend more time in the finishing phase, increasing their susceptibility to heat stress while simultaneously being exposed to challenging environmental conditions. As heat stress in feedyards becomes a greater concern for cattle performance, welfare, and economics, heat stress mitigation strategies have been explored. The National Cattlemen’s Beef Association Beef Quality Assurance program provides useful practices to help prevent heat stress in high-risk groups of cattle, mainly through altering cattle handling and transport in extreme weather [11]. Certain beef cattle certification programs have guidelines to follow to minimize heat stress, including Global Animal Partnership and Humane Farm Animal Care [12,13]. These typically include access to shade and water-cooling systems (sprinklers). Cattle at greater risk for heat stress are generally considered first when prioritizing shade or sprinkler systems. Risk factors for heat stress include dark hide color, prior pneumonia treatment, greater fatness, and excitability [14]; therefore, shade and sprinklers are typically seen in receiving, shipping, and hospital pens. However, because of the cost of shade structures and increased water usage by sprinklers, alternative mitigation strategies warrant investigation.

Elevated body temperature typically leads to a protective lowering of metabolic activity and suppressed appetite [15,16,17,18]. Thermal heat load in cattle is determined by both environmental and metabolic heat production. Ray et al. (1971) [19] noted two major periods of feedlot cattle feed consumption, one at sunrise and one in the afternoon. However, hot summer conditions delayed and decreased time at the bunk in the afternoon, and increased feed consumption later in the evening. Conventionally, feedyard cattle are fed during the morning to early afternoon hours. Thus, peak metabolic heat production as a result of feed consumption occurs at a similar time as the peak environmental heat of the day. Feeding heifers in the afternoon was found to increase heat production in the cooler hours of the day when conduction and radiation were more efficient for dissipating heat [20]. One study found that tympanic temperature decreased when steers were fed in the afternoon (1600 h), compared to the morning (0800 h), when the bunks were empty for several hours before feeding [21]. Alternative ways to help mitigate heat stress include altering dietary components of the ration. Increased beef cattle performance in heat stress conditions have been found with dietary inclusion strategies consisting of linseed oil [22], *Allium mongolicum* Regel powder supplementation [23], wet distillers grains with solubles and zilpaterold hydrochloride [24,25]. Recently published reviews regarding microalgae inclusion into dairy goat diets [26] and dairy cow diets [27] found improved fatty acid profiles of milk produced by both species fed microalgae.

Limited, conflicting studies have been conducted to determine the effect of feeding time on cattle performance. Positive effects of feeding time were observed in Barajas et al. (2013), where they found that feeding 70% of the day’s feed delivery to nonshaded crossbred steers during a period of high ambient temperatures at 1430 h compared to 0630 h increased average daily gain, gain efficiency, and dietary NE [28]; and a study conducted in 1994 found that feedlot limit-fed Holstein steers had greater gain and feed efficiency when fed at 2000 h compared to 0800 h [29]. A study conducted using Hereford steers found that steers with access to feed between 1600 and 0800 h the following day had increased feed intake during a hot period and a lower rectal temperature at 1800 h compared to steers fed ad libitum [30]. Conversely, Mader and Davis did not find differences in Angus yearlings fed in the morning versus the afternoon on ADG during summer months [31]. Regarding dairy cattle, a crossover study, using eight lactating cows, was used to determine if evening feeding (2030 h) would help alleviate associated production losses seen during heat stress events; however, no differences were seen in vaginal temperature, respiration rate, dry matter intake, water intake, or milk production parameters [32]. The authors believe these inconsistent findings are due to the different feeding strategies (e.g., limit-fed/restricted), timing and number of feedings, and the variable heat load and duration within the studies. These inconsistent findings highlight the need for further research, particularly under large-scale commercial conditions.

The objective of this study was to compare performance and behavior outcomes of terminally sorted steers fed in the morning (AM) versus steers fed in the evening (PM) throughout the finishing period and through harvest. We hypothesized that PM-feeding will improve performance (average daily gain, feed conversion, carcass characteristics) and behavior (open mouth breathing prevalence) outcomes by shifting metabolic heat production to cooler periods of the day.

## 2. Materials and Methods

All procedures described within this study were approved by the Animal Care and Use Committee of Kansas State University (IACUC-5251-DFT). Sample size was determined based on an expected difference of 18 KG in final body weight (536 kg vs. 554 kg), derived from Experiment 1 reported by Knutsen et al. [33]. Using an alpha of 0.05 and beta of 0.20 with standard deviation of 20 the number of pens to statistically identify this difference was calculated to be 21 per treatment group. We used 15% more pens (n = 24 per treatment group) in anticipation of potential data loss. Animal enrollment into the study began on 4 June 2025, and the final pens shipped to harvest on 25 September 2025.

### 2.1. Animals and Study Timeline

The location of the commercial feedyard was in the Pacific Northwest region of the United States. In this region, it is dry and arid, with approximately 8–9 inches of annual rainfall and an average high temperature in the month of July around 33 degrees Celsius [34]. The Köppen climate classification describes this area as a cold, semi-arid climate [35]. This randomized controlled trial included native crossbred beef steers scheduled for terminal sort, approximately 60 days from their projected ship date. Terminal sorting allows for a large group of cattle to be sorted into smaller, more homogenous, marketing groups based on body weight and hip measurements. Allocation occurred between 4 June and 20 June 2025. Six pens were enrolled per day (blocks; n = 8), over a total of three weeks, totaling 48 pens. At terminal sort, each steer was reimplanted (Synovex Plus, Zoetis, Parsippany, NJ, USA). Using a sorting system (PenPoint Sort, Elanco Animal Health, Greenfield, IN, USA), body weight and hip measurements were collected to determine the respective terminal sort group for each individual animal; heavy (T3), medium (T2), or light (T1). Within the sorting system, a small variance set in between each sort group to allow for similar head counts. The average body weight and standard deviation for the T3, T2, and T1 groups were 645.01 ± 9.98 kg, 601.56 ± 9.53 kg, and 556.65 ± 8.16 kg, respectively.

After each animal was allocated to their respective sort group in the chute, they were then individually randomized to morning-time fed (AM) or night-time fed (PM) treatment groups using a random number generator (Microsoft Excel, Microsoft Corp, Redmond, WA, USA). Each terminal sort day (block; n = 8), six pens were enrolled: T3 AM (n = 1), T3 PM (n = 1), T2 AM (n = 1), T2 PM (n = 1), T1 AM (n = 1), T1 PM (n = 1), until 48 pens were enrolled (24 AM, 24 PM). Each dirt floor pen allowed approximately 150 animals, with 125 square feet of pen space per head. All pens were fed a finishing diet of flaked corn, high moisture corn, potato byproduct, alfalfa hay, wet distillers grain, liquid finisher, and micro ingredients (56.5% DM, 13.1% DM protein, 62.7 Mcal/cwt net energy for gain) for the duration of the study. Lubabegron (Experior^®^, Elanco Animal Health, Greenfield, IN, USA) was fed for the last 56 days before the pens’ projected ship date, with a wash-out period of at least 3 days before harvest.

On day 0 (day of terminal sort), all AM and PM pens allocated were fed conventionally, with first feeding starting at 0500 h, second feeding at approximately 0800 h, and third feeding at approximately 1200 h. For the remainder of the study, AM pens were fed with this forementioned schedule. On the evening of day 0, PM pens were fed their next day’s feed, with no feed call adjustment. Feeding times for the PM pens were 2000 h, 2300 h, and 0200 h the following morning. This feeding schedule continued for 2 evenings with no feed call adjustments. On the evening of day 3, feed calls for the PM pens were decreased to allow for cattle to consume the leftover feed in the bunks. Because of the nature of different feeding times and bunk conditions, blinding of treatment groups in this study was not possible.

Temperature-humidity index (THI) was recorded every day at 1600 h, using a previously published equation [36]. The THI was then binomialized to <80 or ≥80 THI, signifying potential heat stress effects [37].

### 2.2. Daily Collections

Water consumption was measured at 1500 h daily, from 6 June 2025 to 6 August 2025, by individual water meters attached to each waterer in liters. Water consumed in liters per 454 kg BW was calculated using the daily water meter reading and the average pen weight determined by Microbeef (Micro Technologies, AmerisourceBergen, Amarillo, TX, USA).

Feed delivery was measured daily for the duration of the study through Microbeef, as DM kgs per head delivered. Because of the differences in feed delivery for the PM pens during the transition, a 6-day acclimation period allowed for cattle to transition to the evening feeding before daily feed delivery was recorded.

Health events were recorded for the entire duration of the study through Microbeef, daily, including morbidity pulls, mortality, and culling events.

Open mouth breathing (OMB) behavior was determined daily from 6 June 2025 to 6 August 2025, at 1600 h, using a scan sample approach by taking a picture from the center of the front of the pen (feed bunk). Each pen was randomized to one of four days, allowing for OMB to be recorded every 4 days for each pen (12 pens/day). Pictures were then analyzed by one trained, blinded observer to determine the total number of heads seen in the pen, standing, with their faces clearly visible to determine open mouth breathing status. Then, the number of cattle that met the case definition of OMB (standing, mouth open, saliva present, and neck extended) were recorded.

### 2.3. Carcass Collections

Pens shipped to harvest between 5 August 2025 and 25 September 2025. Paired pens (AM and PM pen from same terminal sort group and block) were always shipped on the same week. Individual carcass reports were received from the packing plant and matched to the individual IDs and pen numbers at enrollment. Average pen hot carcass weight (HCW) was recorded using the carcass reports, and an assumed 62.5% dressing percentage was used to determine the approximate final live weight (FLW) [38]. The number of cattle that were graded in each quality grade category (prime, choice, select, other) and yield grade category (1, 2, 3, 4, 5) were recorded, as well as the total number of individual carcass reports received for each pen. ADG was calculated by dividing the difference between FLW and enrollment weight by DOF. Feed conversion (F:G) was calculated by dividing the total DM feed delivered by the total weight gain of the pen.

### 2.4. Statistical Design and Analysis

Pen served as the experimental and observational unit for all analyses except water consumption. A waterer was shared between two pens; therefore, the pair of pens served as the experimental and observational unit for water consumption. A significance level was set at *p* ≤ 0.05, and tendencies at 0.05 < *p* ≤ 0.10. All statistical analyses were performed using R Studio (version 2026.01.0+392, PBC, Boston, MA, USA). Normality of continuous outcomes was assessed using the Shapiro–Wilk test and visualization of histograms.

Continuous outcomes (water consumption, feed delivery, FLW, HCW, ADG, F:G) were analyzed using linear mixed-effects models assuming normally distributed residuals and using an identity link function. Fixed effects included treatment group. Random effects included terminal sort group nested within block to account for lack of independence among observations. For repeated-measures outcomes (water consumption and feed delivery), THI and the potential interaction between treatment group and THI were included as additional fixed effects, and paired pen or pen number was included as an additional random effect. A repeated covariance structure was used, including calendar date to account for serial dependence. For the water consumption model, THI of the observed day was used and sort group nested within block was not included as a random effect as both pens sharing a water could come from different sort groups or blocks, as long as they were the same treatment. For the feed delivery model, THI recorded at 1600 h on the previous day was used because feed calls were based on the previous day’s consumption.

Health, behavior, and carcass grade (quality and yield grade) outcomes were analyzed using generalized linear mixed-effects models, with events over trials as the outcome. Morbidity, mortality, culling, OMB prevalence, quality grade, and yield grade counts were analyzed assuming a binomial distribution and a logit link function. Terminal sort group nested within block was included as a random effect, and pen was included as an additional random effect to account for repeated measures in OMB prevalence. A repeated covariance structure was used for the OMB model, including calendar date to account for serial dependence.

## 3. Results

A total of 7848 native crossbred beef steers were enrolled in this trial. At enrollment, steers were randomized, based on terminal sort group (n = 3), to either AM or PM pens. Table 1 shows central tendencies and variability between terminal sort groups and treatment groups based on number of heads per pen and average body weight per head at enrollment.

**Figure 1:**
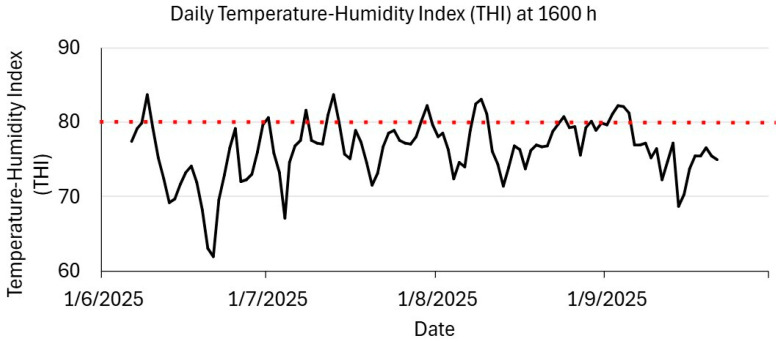
Temperature-humidity index (THI) recorded at 1600 h from 6 June 2025 to 25 September 2025. A red dotted line depicts the threshold of 80 THI, the case definition for a potential heat stress event.

Figure 1 depicts the THI recorded at 1600 h over the entire study duration. A dotted red line differentiates the threshold of 80 used in statistical analyses. Sixteen days (14%) were recorded as having a THI ≥ 80, with a maximum recorded THI of 84.

The PM-fed pens were successfully transitioned to night-time feeding using the methods described above. By day 6 after pen enrollment, the feed calls made to the PM pens matched those being made for the AM pens without any numerical increases in digestive morbidity or mortality.

Performance and behavioral variables were generally similar between treatments throughout the study period. Table 2 describes model estimated means of daily collections (water consumption, feed delivery, OMB); however, treatment group was not significant for any outcomes (*p* > 0.05). Overall health (morbidity, mortality, culling), performance (final live weight, daily gain, feed efficiency), and carcass (hot carcass weight, quality grade, yield grade) outcomes are shown in Table 3. No differences (*p* > 0.05) were seen between treatment groups for any outcome.

Temperature-humidity index (THI) was found to have a tendency and significant difference in regard to daily water consumption rate and OMB, respectively, regardless of treatment group (Table 2). When the THI at 1600 h was ≥80, all cattle tended (*p* = 0.07) to consume more water and had a higher probability (*p* < 0.01) of OMB than on days with a THI < 80, indicating marginal effects of heat stress occurring between both treatment groups.

## 4. Discussion

The aim of this study was to compare performance and behavior outcomes of terminally sorted steers fed in the morning (AM) versus steers fed in the evening (PM) throughout the finishing period and through harvest. Steers were transitioned to the PM-feeding schedule successfully; however, no detectable differences were seen between treatment groups. Regardless of AM- or PM-fed group, cattle seemed to respond to the effects of heat stress, and management practices should be put in place to mitigate these effects.

As a result of limited research published on transitioning fattening cattle from morning- to evening-fed, study personnel consulted multiple industry personnel (veterinarians, nutritionists, feedyard managers) to gauge opinions on how to successfully transition the study steers. In this study, steers were transitioned to PM-feeding by feeding the next day’s ration the evening before and not changing the feed call for three days, allowing steers to consume their desired amount of feed at any time of the day. This prevented the bunk from becoming empty to avoid increasing the risk of acidotic events seen commonly with fluctuating feed consumption. On the fourth evening after enrollment, feed delivery was reduced to allow steers to consume the remaining feed in the bunk. By day 6, the PM pens’ feed delivery was consistent with the AM pens within the same block. With this schedule, the PM steers’ transition was achieved without observable digestive morbidity or mortality.

In the current study, no statistically significant differences were found between treatment groups for any outcome. Due to limited research on evening feeding, specifically regarding the capacity of a commercial feedyard with many cattle in each pen, authors compared the variation seen in the current study to other published studies. Ominski et al. (2002) had greater variation around dry matter intake and water consumption [32]. Brandt and Reinhardt (1994) found lesser variation compared to the current study regarding daily gain, but a greater variation in terms of feed efficiency [29]. Mader and Davis (2004) had greater a variation compared to the current study regarding water intake, similar variation on overall daily dry matter intake, and lesser variation regarding feed efficiency compared to the current study, with no statistical differences found between treatment groups [31]. Because of the variation between studies, we believe that the lack of differences detected in this study may have been due to having few days with high THI associated with heat stress. It has been shown that metabolic heat production produced by microbial fermentation accounts for 3 to 8% of the total heat production of cattle [39]. During increased environmental conditions, cattle compensate by eating smaller meals, more frequently, and shifting feed intake to cooler parts of the day [19,40,41]. The lack of differences seen in this study could be a result of the AM-fed cattle adjusting their feed consumption naturally. Another contributing factor may be that only 14% of the total study days had a THI ≥ 80, with the maximum THI equaling 84. Due to the increased THI occurring in the middle of the summer, cattle may have had substantial time to acclimate to the increased THI. Other weather variables like windspeed, solar radiation, and night-time temperatures also play a role in thermal load, but were not recorded in the present study. The current study was conducted at a larger scale than many previous experiments, involving approximately 7800 steers across 48 pens. This increase in scale more closely reflects commercial feedyard production conditions and may aid in explaining discrepancies between our findings and earlier studies conducted under smaller experimental settings. The present study was the first to feed three feedings throughout the night-time hours, rather than the late evening hours seen in previous studies. Differences in feeding strategies (timing and number of feedings) could result in the differences seen regarding outcomes.

Regardless of treatment groups, when the THI crossed the threshold of 80, water consumption and OMB prevalence increased, indicating heat stress in the cattle. The increase in water consumption and panting behavior is commonly seen under heat stress conditions [6,8]. In just the summer months alone, Arias and Mader (2011) reported a 87.3% increase in water consumption compared to winter months in feedlot cattle [42]. Idris et al. (2024) found that respiration rate and panting score nearly doubled in heat stress conditions compared to thermoneutral conditions [8]. Heat stress mitigation strategies should continue to be explored and implemented by producers, including the use of shade or water-cooling systems. Further research is warranted on the effects of evening feeding on feedyard cattle performance, enabling producers to determine if this strategy can be effectively implemented either independently or in combination with other heat stress mitigation practices.

Limitations to this current study include the lack of extreme heat (days with THI ≥ 80 at 1600 h) and the inability to blind to treatment group. Due to time constraints of capturing OMB prevalence daily, 12 pens per day were observed within a similar environmental time frame. Authors do recognize that short-term fluctuations in OMB within pens may not be captured with this data collection strategy; however, the same number of pens per treatment were recorded daily. Possible confounding effects associated with different feed callers between the AM and PM group could have influenced feed delivery; however, all feed callers were trained using standardized company procedures. 

## 5. Conclusions

Night-time feeding was successfully implemented in a commercial feedyard, demonstrating that this management strategy is feasible under practical conditions. However, no detectable differences were found between the morning and evening feeding schedules across all measured outcomes, possibly due to relatively mild environmental conditions during the study period. Despite the lack of treatment differences, cattle were shown to increase water consumption and open mouth breathing prevalence, indicating some effect of heat stress when the temperature-humidity index was greater than or equal to 80. This emphasizes the continued importance of heat stress mitigation strategies within the feedyard sector. The implementation of evening feeding is feasible, but not sufficient alone under mild heat stress conditions. Further research is warranted to evaluate the effects of evening feeding under more severe or prolonged environmental conditions, as well as in combination with other heat stress mitigation strategies, such as shade or cooling systems, to better determine its potential role in improving cattle productivity and welfare.

## Figures and Tables

**Table 1:** Pen demographics at enrollment of steers allocated at terminal sort (T1: small; T2: medium; and T3: big) and randomized to either AM-fed (AM) or PM-fed (PM) treatment groups.

		Treatment Group			
		AM		PM	
	Terminal Sort Group	Mean	SD	Mean	SD
Total head per pen (n)	T1	163	10	164	11
	T2	165	9	164	11
	T3	162	10	163	11
Average bodyweight per head (kgs)	T1	555.52	8.64	556.11	8.31
	T2	601.20	10.55	599.94	9.34
	T3	645.53	9.06	644.57	11.87

**Table 2:** Treatment (trt) and temperature-humidity index (THI) model estimated daily outcomes of terminally sorted steers randomized to AM-feeding (AM) or PM-feeding (PM) during a heat stress trial in the summer of 2025 at a commercial feedyard in the Pacific Northwest.

	Model Estimated Means ± SE						
	Trt		THI		*p*-Value		
Daily Outcomes:	AM	PM	<80	≥80	Trt	THI	Trt·THI
Water consumption (L/454 kgs BW)	31.38 ± 0.55	31.99 ± 0.60	31.15 ± 0.33	32.18 ± 0.63	0.14	0.07	0.40
Feed delivery (kgs DM/head)	10.8 ± 0.14	10.9 ± 0.14	10.8 ± 0.13	10.9 ± 0.13	0.17	0.26	0.71
Open mouth breathing (% pen)	0.6 ± 0.3	0.4 ± 0.2	0.2 ± 0.09	1.5 ± 0.78	0.66	<0.01	0.67

**Table 3:** Treatment model estimated overall outcomes of terminally sorted steers randomized to AM-feeding (AM) or PM-feeding (PM) during a heat stress trial in the summer of 2025 at a commercial feedyard in the Pacific Northwest.

	Treatment Model Estimated Means ± SE		
Overall Health Outcomes:	AM	PM	*p*-Value
Morbidity (n treatment/hd count)	13 ± 1	14 ± 2	0.31
Mortality	1 ± 0.3	1 ± 0.3	0.77
Culling	0.6 ± 0.2	0.5 ± 0.1	0.50
Overall performance and carcass outcomes:			
Final live weight (kgs)	775 ± 5.17	774 ± 5.17	0.83
Hot carcass weight (kgs)	484 ± 3.23	484 ± 3.23	0.83
Daily gain (kgs)	2.1 ± 0.03	2.2 ± 0.03	0.38
Feed:gain (DM/kgs)	4.99 ± 0.08	4.92 ± 0.08	0.30
Quality grade prime and choice (% carcasses)	97 ± 0.3	97 ± 0.4	0.34
Yield grade 1, 2, 3 (% carcasses)	69 ± 1	70 ± 1	0.42

## Data Availability

The original contributions presented in this study are included in the article. Further inquiries can be directed to the corresponding author.

## Abbreviations

The following abbreviations are used in this manuscript:

PM	Evening feeding
AM	Morning feeding
THI	Temperature-humidity index
OMB	Open mouth breathing
HCW	Hot carcass weight
FLW	Final live weight
F:G	Feed conversion; feed:gain
ADG	Average daily gain
